# Quantitative evaluation of an innovation contest to enhance a sexual health campaign in China

**DOI:** 10.1186/s12879-019-3746-4

**Published:** 2019-02-04

**Authors:** Ye Zhang, Songyuan Tang, Katherine Li, Lai Sze Tso, Barry L. Bayus, David Glidden, Bin Yang, Heping Zheng, Chongyi Wei, Joseph Tucker, Weiming Tang

**Affiliations:** 1 Dermatology Hospital, Southern Medical University, Guangzhou, China; 2 University of North Carolina Project-China, No. 2 Lujing Road, Guangzhou, 510095 China; 3 Kirby institute, University of New South Wales, Sydney, Australia; 4 School of Public Health, Kunming Medical University, Kunming, China; 5 Weill Cornell Medical College, New York, NY USA; 6 Kenan-Flagler Business School, University of North Carolina, Chapel Hill, NC USA; 7 Department of Epidemiology and Biostatistics & Global Health Sciences, University of California, San Francisco, CA USA

**Keywords:** Innovation contest, Crowdsourcing, Public health campaign, Sexual health

## Abstract

**Background:**

Crowdsourcing method is an excellent tool for developing tailored interventions to improve sexual health. We evaluated the implementation of an innovation contest for sexual health promotion in China.

**Methods:**

We organized an innovation contest over three months in 2014 for Chinese individuals < 30 years old to submit images for a sexual health promotion campaign. We solicited entries via social media and in-person events. The winning entry was adapted into a poster and distributed to STD clinics across Guangdong Province. In this study, we evaluated factors associated with images that received higher scores, described the themes of the top five finalists, and evaluated the acceptability of the winning entry using an online survey tool.

**Results:**

We received 96 image submissions from 76 participants in 10 Chinese provinces. Most participants were youth (< 25 years, 85%) and non-professionals (without expertise in medicine, public health, or media, 88%). Youth were more likely to submit high-scoring entries. Images from professionals in medicine, public health, or media did not have higher scores compared to images from non-professionals. Participants were twice as likely to have learned about the contest through in-person events compared to social media. We adapted and distributed the winning entry to 300 STD clinics in 22 cities over 2 weeks. A total of 8338 people responded to an acceptability survey of the finalist entry. Among them, 79.8% endorsed or strongly endorsed being more willing to undergo STD testing after seeing the poster.

**Conclusions:**

Innovation contests may be useful for soliciting images as a part of comprehensive sexual health campaigns in low- and middle-income countries.

**Electronic supplementary material:**

The online version of this article (10.1186/s12879-019-3746-4) contains supplementary material, which is available to authorized users.

## Background

Sexual health has become an increasingly relevant and controversial topic among youth in China. More and more Chinese, particularly Chinese youth, have adopted more open attitudes toward sex and are engaging in premarital sex [1, 2]. However, sexual education in China has not evolved to meet their needs, and many young people have limited knowledge of safe sex. Surveys of adolescents and college students have shown high rates of condomless sex and limited knowledge about STD transmission and reproductive health [3–8].

Recently, researchers have implemented community outreach programs that have shown success in improving sexual health knowledge [9–11]. However, these programs can be expensive. Further, they are typically designed in a top-down approach by public health organizations or media companies without input from the community. As a result, young people may not feel comfortable or motivated to engage with them due to social stigma and differences in cultural understanding.

Innovation contests may be an effective approach to limit costs while improving engagement with sexual health campaigns. Innovation contests call upon crowd or non-expert wisdom to accomplish a task or generate new ideas [12, 13]. These contests have been used in business and computer science [14–17], as well as in public health [12, 13, 16, 18–23]. Health-focused innovation contests have increased knowledge [18, 24], reduced intervention costs [23], and improved access to health information in high-income countries [25].

However, very few studies have rigorously evaluated innovation contests in low and middle-income countries [12, 26]. Our previous research showed that an innovation contest was as effective as a social marketing approach in designing an HIV testing campaign [27]. The innovation contest also significantly decreased campaign cost and effectively engaged local communities [24].

We conducted an image contest in 201 which has described in detail elsewhere. [12]. The purpose of this contest was to solicit image submissions to promote sexual health. This study aims to evaluate the themes of the top five entries, examine correlates of higher quality images, and assess the acceptability of the winning image produced by the contest.

## Methods

The crowdsourcing contest described in this study was conducted in 2014, while the online survey mentioned in this study was conducted in 2015.

### Innovation contest

The overall contest last for 3 months, including the promoting campaign and 7 weeks the effective time for sending the entries. Our target audience for contest participation consisted of people in the community younger than 30 years old. First, we started and promoted the contest through an open call for images promoting sexual health on social media platforms. This included a series of posts on WeChat (a mobile text and voice messaging communication service in China, which is a Chinese hybrid between Facebook and Twitter) and Weibo (a China-based social networking and microblogging service websites, similar to Twitter). Posts were included on social media feeds of organizations focused on sexual health (e.g., lesbian, gay, bisexual, and transgender (LGBT) organizations, HIV community groups). Next, to further reach the target population, we hosted in-person events on college and high school campuses as well as the US Consulate in Guangzhou. Guangdong provincial is one of the biggest province in China with 109.7 million residents in 2017, and among of them 81.5 million people is around 18–60 years old. These included classroom presentations at universities in Guangzhou, interactive feedback sessions at the US Consulate in Guangzhou, and outreach at community-led activities.

Classroom presentations were instructional sessions led by contest organizers to present the contest mission and criteria as well as to answer sexual health questions. Interactive feedback sessions were activities implemented by contest organizers along with community-based organizations and student groups to provide feedback about potential entries prior to submission. Outreach at community-led activities consisted of a brief announcement and call for submissions given by contest organizers at events held by local community-based organizations, such as speed dating or matchmaking (see Additional file 1: Table S1).

The incentives to promote contest participation included a range of prizes (First prize: 750 USD in cash, second prize: a Kindle (about 150 USD), third prize: a camera (about 60 USD)). Furthermore, final entries would be displayed in an open entry photograph gallery in Guangzhou.

Judging was conducted in two phases. The main criteria were 1) relevance to sexual health promotion and 2) capacity to interest youth in China. First, each eligible image was independently assessed by three non-expert voluntaries which were recruited through WeChat. Then, we recruited 18 expert judges in the fields of medicine, public health, and media through local contacts and community-based organizations. The remaining images were independently evaluated on a scale of 0–10 by three of the expert judges. Personal identifiers were removed from all images to prevent bias. The mean score from the three judges was used to rank the submissions. Finally, we celebrated the submissions and community contributions through an interactive award ceremony on World AIDS Day in 2014 [12].

### Participant demographics and data collection

Individuals living in China aged 30 and under were eligible to participate in the image contest. All participants submitted demographic details upon submission, including age, the province of residence, means of learning about the contest (i.e., through social media or in-person events), whether or not they were experts in public health, medicine, media, or related fields.

### Campaign implementation

Following the conclusion of the contest, we obtained permission from winner to use the winning submission. This submission was adapted for local intervention campaigns for promoting sexual health in Shandong and Guangdong provinces. Additionally, the winner also redesigned the finalist entry as a poster which was displayed in 300 STD clinics in 22 cities in Guangdong province over the course of two weeks. The poster was also displayed on the SESH website and SESH WeChat moment.

### Online poster evaluation survey

Each poster of the winning entry contained a QR code which led to an online acceptability survey, which was open for one month. Anyone who saw a poster could scan the QR code and participate in the survey. Each participant’s IP address and WeChat ID was recorded, so that each person could only submit the survey once. To be eligible for the survey, the participants had to be at least 15 years of age. At the end of the survey, each participant could play an online raffle game to earn 0 to 1 USD, which served as an incentive for survey participation.

The goal of the survey was to evaluate the acceptability of the single finalist entry and to collect feedback on the poster from the viewers. The survey contained questions about the demographic information of participants, their attitudes toward the poster, and their willingness to have more STD/HIV related knowledge, and their willingness to have an STD/HIV test after seeing the poster. The response options consisted of a 5-point Likert Scale, where 1 was indicated as strong disagreement and 5 was defined as strong agreement. Participants could also forward the survey to their friends.

### Analysis

Data were cleaned and recoded using Microsoft Excel 2008 and analyses were performed using SPSS 21.0. We coded several characteristics of the submissions: multi-panel (consisting of multiple images in a single submission) or not, photography or not, drawn image or not, language used (Chinese, English, or both), and timing of submission. We then used descriptive analysis to analyze socio-demographic characteristics of the contest submitters and characteristics of the submissions, including the language used, drawn images versus photography, and a presence of text. We also described the themes, messages, and images on the top five contest submissions.

Multivariable linear generalized estimating equation (GEE) with unstructured correlation matrix was used in order to evaluate the correlates associated with higher image scores [28]. We used a mixed-effects linear regression model to adjust the non-zero-image scores by accounting for heterogeneity of scores among judges. Then we used the paired Wilcoxon signed-rank test to compare the difference between median adjusted and unadjusted scores. Lastly, we used descriptive analysis to evaluate the attitudes of the STD clinics attendees towards the poster as well as the effectiveness of the poster in improving their knowledge of STDs and their willingness to pursue future STD/HIV testing.

## Results

### Characteristics of image contest participants and entries

We received 96 entries over 43 days from 76 participants across 10 provinces and province-level cities in China. Sixty-five (85.5%) participants were from Guangdong province and eleven (14.5%) were from outside Guangdong province (Table 1). Eleven participants (14.0%) submitted multiple entries while 65 participants (86.0%) submitted one entry. The median age of participants was 20.0 (18/23.5) years old, while 85% of them were under 25 years old. Nine (11.8%) participants were self-reported healthcare/public health/design/media professionals, while six (7.9%) were employed full-time in a public health, media or a related field.

**Table 1:** Social and Demographic Characteristics of Contest Participants and Characteristics of Images from Sexual Health Image Contest, China 2014

Variables	No. of Individuals/images	(%)
Social and Demographic Characteristics of Contest Participants(*n* = 76)		
Age		
Mean ± SD [Range]	20.0 ± 3.5 [16–30]	
Location		
Guangdong Province	65	(85.5)
Outside Guangdong Province	11	(14.5)
Sexual health expert or not^a^		
Yes	9	(11.8)
No	67	(88.2)
Attended in-person event^b^		
Yes	57	(75.0)
No	19	(25.0)
Aware of contest through		
Weibo	5	(6.6)
WeChat	10	(13.2)
Friend	18	(23.7)
In-person events	40	(52.6)
Others	3	(3.9)
Willing to hear about future contests		
Yes	51	(67.1)
No	25	(32.9)
Characteristics of Images (*n* = 96)		
Average score		
Mean ± SD	4.53 ± 2.44	
Language used		
Chinese	49	(51.0)
English	30	(31.3)
English +Chinese	17	(17.7)
Photo image		
Yes	20	(20.8)
No	76	(79.2)
Multiple panels		
Yes	80	(83.3)
No	16	(16.7)
Drawn images		
Yes	45	(46.9)
No	51	(53.1)
Words included or not		
Yes	67	(69.8)
No	29	(30.2)

^a^Sexual health expertise was defined as whether or not participant self-identified as an expert in public health, medicine, or media

^b^Attendance at in-person events included participating in classroom didactics, interactive feedback sessions, and community-led activities. Classroom didactics were instructional sessions led by contest organizers to present on the contest mission, criteria, and to answer sexual health questions. Interactive feedback sessions were activities implemented by contest organizers via consultation with community-based organizations and student groups to give feedback about potential entries prior to submission. Community-led activities were implemented jointly by local community-based organization in collaboration with the contest organizers

Seventy-five percent of participants attended in-person events hosted by our team or a community partner (Table 1). Participants most often heard about the contest through in-person events (*n* = 40, 52.6%), followed by friends (*n* = 18, 23.7%), WeChat postings (*n* = 10, 13.2%), Weibo (*n* = 5, 6.6%), and other methods (*n* = 3, 3.9%) (Table 1).

The mean overall score of the entries was 4.5 with a standard deviation of 2.4 and a range of 0 to 8.9. Of the 96 images, 49 (51.0%) used only Chinese language, 30 (31.3%) used English only and 17 (17.7%) used both. Sixty-seven (69.8%) submissions used a combination of words with images. Forty-five (46.9%) submissions contained a drawn image. Overall, around four-fifths (83.3%) of images contained multiple panels (a single entry with a series of two or more images). 65 images (67.7%) were submitted during the final week of the contest.

### Description of the top five entries of the image contest

Table 2 presented the top five entries of the image contest. Within the top five entries (scored as top five by the judgers), No. 1 and No.2 entries focused on the intersection of sex, health and love, while the other three focused on promoting condom use during sex. In addition, two of the top five entries used more than one image. No. 4 and No.5 entries designed a slogan promoting responsible sexual health behaviors with their images; No.5 used Photoshop to create an image of a condom packet that resembled a lock and featured the slogan “lock your health”.

**Table 2:**
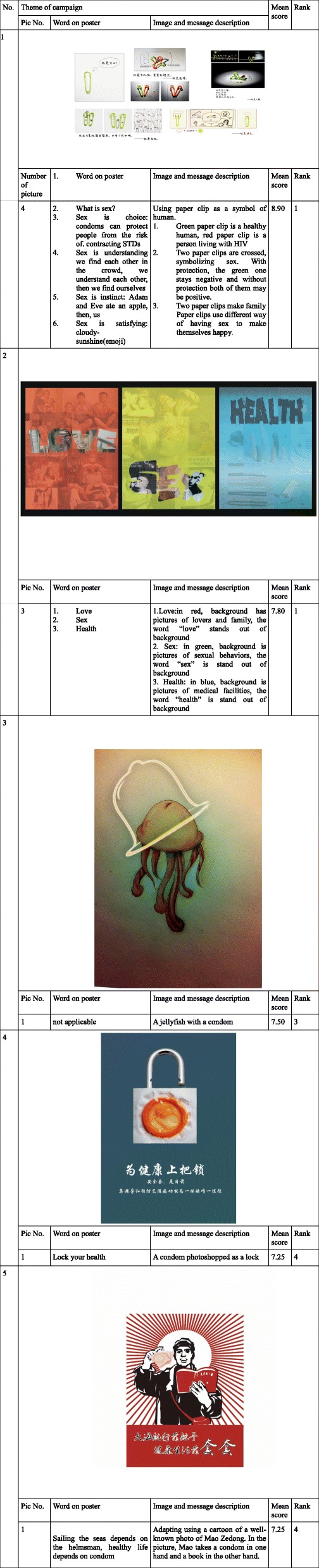
Themes developed by Images from Sexual Health Image Contest, China 2014

### Correlates of image quality

To evaluate the correlates associated with higher image scores, our multivariable GEE analysis showed that youth < 25 years old submitted higher quality images (B = − 0.16, 95% CI: -0.26, − 0.03, Table 3). Higher quality images were also associated with having attended in-person events (B = 1.29, 95% CI: 0.29, 2.29). Images generated by non-experts were not significantly different in comparison to images created by experts (B = 1.23, 95% CI: -0.56, 3.03). Nine of the top ten highest scoring images were created by non-experts. (Table 3).

**Table 3:** Summary of Multivariate GEE Analyses: Final Score by Socio-demographics Characteristics of Submitters and Characteristics of Images, China 2014 (*n* = 96)

	Wald Chi-square	B (95% CI)	*P* value
Age	5.769	− 0.16 (− 0.26, − 0.027)	0.016
Attended in-person event			
No	Ref	Ref	Ref
Yes	6.389	1.29 (0.29, 2.29)	0.01
Sexual health experts or not			
Yes	Ref	Ref	Ref
No	1.809	1.23 (0.56, 3.03)	0.179
Photo image			
Yes	Ref	Ref	Ref
No	28.181	3.15 (1.99, 4.32)	< 0.001
Drawn image			
No	Ref	Ref	Ref
Yes	4.565	0.96 (0.079, 1.84)	0.033

### Acceptability of the single finalist entry from an online survey

Overall, 8343 respondents participated in the online evaluation survey. The ages of participants ranged from 16 to 80 years, with a mean age of 28.0 ± 3.5. Among them, 2665/8343 (31.9%) of respondents saw the finalist poster in an STD clinic and the remaining (68.1%) saw the finalist poster online. The single finalist entry is shown in Table 2 (first figure).

The results of the Likert Scale evaluation showed that nearly half of participants (43.0%) strongly liked the poster with the winning image, 54% participants liked the poster, and only 3% participants disliked or strongly disliked the poster. The majority of respondents reported that they would be likely or very likely to obtain more information about STD after seeing the poster (85.2%). Furthermore, 43.2% of participants reported that they would be very likely to go for STD testing after seeing this poster, 36.3% participants would be likely to do so, and only 7.5% participants would be unlikely or very unlikely to do so (Table 4).

**Table 4:** Summary of Likert Scale evaluation from an online survey in China, 2016 (*N* = 8338)

Variables	Strongly endorse	Endorse	I don’t know	Do not endorse	Strongly do not endorse
Attitude towards the poster	3585(43.0%)	3622(43.4%)	876(10.5%)	186(2.2%)	69(0.8%)
Willingness of knowing more STD related knowledge	4003(48.0%)	3028(36.3%)	932(11.2%)	270(3.2%)	105(1.3%)
Willingness of doing STD/HIV test	3598(43.2%)	3052(36.6%)	1062(12.7%)	410(4.9%)	216(2.6%)

The online survey also collected open-ended feedback from participants. Recurrent themes in feedback included: 1) the graphic on the poster was innovative, attractive at first glance, and interesting; 2) the poster was thought provoking; 3) the poster could be improved through using fewer words; 4) the subject of the poster was too general; and 5) the poster could be put in public places rather than only in STD clinics.

## Discussion

Our study evaluated the quality, characteristics, and acceptability of sexual health campaign images solicited by an innovation contest. Previous literature has shown that innovation contests may be useful in promoting sexual health campaigns. [18, 24] Previous work from our group has shown that implementing an innovation contest for a sexual health campaign effectively improved community engagement and resulted in a more patient-focused campaign ^17^. Our work confirms the utility and effectiveness of innovation contests in sexual health campaigns, and expands the existing literature by examining qualities of the finalist entries, quantitatively exploring correlates of high-quality entries, and evaluating the acceptability of the winning entry through an online public survey.

Our study found that youth under age 25 were more likely to submit high-quality entries compared to older individuals (age 25–30) in this our contest. This finding is consistent with other contest literature showing high-quality youth participation [29]. Previous research has examined youth participation in business contests [29] and assessed the impact of youth as peer counselors for HIV/STD interventions [30–34]. Our research further supports the importance of engaging youth in sexual health efforts. We also found that youth who actively attended in-person events were more likely to submit high-quality images and join our celebratory activities. This highlights the importance of holding in-person events in future innovation contests.

Importantly, we found that there was no difference between scores of experts (i.e. employed in the fields of medicine, public health, or media) and non-expert entries. Nine out of the top ten entries were submitted by non-experts. This is consistent with the concept of crowd wisdom [35, 36] and a broader business literature showing how non-expert work can generate innovative, useful ideas [17, 21]. This finding supports the use of innovation contests to engage non-experts in sexual health campaigns [37–40]. Future qualitative research would be helpful in understanding how to distribute campaign responsibilities between experts and non-experts.

The winning image from our contest was successfully scaled up and displayed in 121 public STI clinics as part of a province-wide sexual health campaign over the course of two weeks. The majority of the survey respondents found the winning image acceptable and engaging, with 43% of respondents strongly endorsing and 43.4% of respondents endorsing approval of the image. Additionally, 43.2% of respondents strongly endorsed and 36.6% of patients endorsed a greater likelihood to seek out STD testing after seeing the winning image. One potential explanation for high acceptability of the final image is that the use of a community-based crowdsourcing approach solicits inclusion of different perspectives, and may often be more in tune with the perspectives of lay persons. Sexual health campaigns are inherently difficult to carry out due to associated cultural stigma and fear of discrimination, and innovation contests can help solicit campaign messages that are culturally sensitive and acceptable. The feedback given on the survey further supported this notion, as many viewers indicated that the crowdsourced images made a sensitive topic more approachable [41].

Our study also supports the existing literature on the efficiency of innovation contests. Previous studies have indicated that innovation contests can generate cost-effective solutions for interventions with quick turn-around times [23]. In this image-generation contest, we successfully crowd-sourced around 100 images from participants in a one-month period, and we implemented the winning entry throughout STD clinics in Guangdong province within 2 weeks. This ability to generate multiple campaign options on an expedited schedule is highly advantageous for the development of effective and timely outreach campaigns for public health. Partnering with community organizations also allows knowledge of the campaigns to spread throughout the community, thereby enhancing the campaign’s reach both before and after implementation. Finally, innovation contests are easy to organize and modify for applications in multiple health campaigns, and they require much fewer resources than traditional social marketing or social media campaigns [23].

There were several limitations to our study. First, all in-person events were held in urban centers in Guangdong province. Generally, young people in urban centers are technologically savvy and eager to engage with community campaigns. We received much fewer submissions from rural areas, and while increasing in-person events in rural settings may improve engagement, it is also possible that young people living in rural areas may not have comparable resources or interest in participating in an innovation contest. Secondly, Guangdong also has a high burden of STDs [42]. Despite limited sexual health education for young people, its residents generally hold more liberal views of sex than residents of other provinces [43]. Thus, young people might feel more comfortable and motivated to engage with an innovation contest in Guangdong as compared to other provinces. Third, the results of our acceptability survey are only descriptive. We did not evaluate the response to the winning campaign images against a campaign image developed by a traditional social marketing company. And finally, the results of our acceptability survey provide only the opinions of the respondents, and not their behaviors. We did not measure the rate of STD testing to evaluate if the campaign materials influenced their viewers’ decisions to proceed with STD testing. Future work should compare the campaign solicited by an innovation contest with a campaign designed by a traditional social marketing agency, as well as evaluate any behavioral changes (i.e. increase in STD testing) after exposure to an innovation contest-produced sexual health campaign.

## Conclusions

Our study raises several policy and public health implications for HIV/STD prevention and awareness campaigns. It advocates for engaging youth in the community. Further, our study showed that in-person events, in addition to social media events, are important facilitators for generating higher-quality solutions. Future sexual health campaigns should incorporate face-to-face interactions where participants can ask questions and solicit feedback about their submission ideas. Importantly, submissions from non-experts accounted for the majority of the top ten entries, highlighting the utility of engaging non-expert audiences. Our survey of the winning campaign image also showed high acceptability of the campaign and a high self-reported rate of increased likelihood to pursue STD testing after viewing the campaign. Additional research comparing outcomes from the campaigns from innovation contests and traditional social marketing is needed to demonstrate the effectiveness of innovation contests. These studies would bolster confidence on the application of innovation contests to future sexual health campaigns.

## Additional file

Additional file 1: **Table S1.** In-person Activities Promoting Sexual Health Image Contest, Sept-Nov 2014, China. (DOCX 19 kb)

## Abbreviations

HIV: Human Immunodeficiency virus
LGBT: Lesbian, gay, bisexual, and transgender
STD: Sexually transmitted diseases
USD: US dollars
