# Supplementary material for: Quantitative evaluation of an innovation contest to enhance a sexual health campaign in China
Source: BMC Infect Dis. 2019 Feb 4;19:112. doi: 10.1186/s12879-019-3746-4 (PMC6360679; doi:10.1186/s12879-019-3746-4)
Supplement: Supplementary file 1 — Table S1. In-person Activities Promoting Sexual Health Image Contest, Sept-Nov 2014, China. (DOCX 19 kb) [file 12879_2019_3746_MOESM1_ESM.docx]

| **Supplemental Table 1. In-person Activities Promoting Sexual Health Image Contest, Sept-Nov 2014, China** | | | | | |
| --- | --- | --- | --- | --- | --- |
|  | Days from contest start | Event date | Host organization | Event type | Activity description |
| A | (Preview 19 days) | Wednesday  09.17.2014 | Medical university in Guangzhou | Community-led activity* | Student group viewed internally made video promoting contest |
| B | Day 3 | Wednesday  10.08.2014 | Comprehensive university in Guangzhou | Classroom didactic** | Five-minute in-class presentation to students by our outreach staff |
| C | Day 14 | Sunday  10.19.2014 | Student group within comprehensive university in Guangzhou | Interactive feedback session*** | All-day information booth with Q&A using posters by our staff |
| D | Day 18 | Thursday  10.23.2014 | Foreign Consulate in Guangzhou | Classroom didactic | 1-hour-30-minute presentation by our staff about our contest/ mission |
| E | Day 24 | Wednesday  10.29.2014 | Pharmacy university in Guangzhou | Interactive feedback session | Information booth outside school cafeteria by our staff (11 am- 6 pm) |
| F | Day 30 | Tuesday  11.04.2014 | International high school in Guangzhou | Classroom didactic | Give 40 minutes presentation on sexual health across five classes |
| G | Day 33 | Friday  11.07.2014 | Comprehensive university in Guangzhou | Community-led activity | Internal announcement created by student group |
| H | Day 37 | Tuesday  11.11.2014 | Student group within comprehensive university in Guangzhou | Community-led activity | Sexual health slogan campaign created and promoted internally by student group with feedback from our staff |

*Community-led activities were defined and implemented jointly by local community-based organization in collaboration with the contest organizations.

**Classroom didactics were instructional sessions led by our team to present on the contest mission, criteria, and to answer sexual health questions.

***Interactive feedback sessions were implemented by our team via consultation with community-based organizations and student groups to give contest

participants feedback about their potential entries.
